# Fano Factor: A Potentially Useful Information

**DOI:** 10.3389/fncom.2020.569049

**Published:** 2020-11-20

**Authors:** Kamil Rajdl, Petr Lansky, Lubomir Kostal

**Affiliations:** Laboratory of Computational Neuroscience, Institute of Physiology, Academy of Sciences of the Czech Republic, Prague, Czechia

**Keywords:** Fano factor, spike trains, variability measure, intensity, renewal process

## Abstract

The Fano factor, defined as the variance-to-mean ratio of spike counts in a time window, is often used to measure the variability of neuronal spike trains. However, despite its transparent definition, careless use of the Fano factor can easily lead to distorted or even wrong results. One of the problems is the unclear dependence of the Fano factor on the spiking rate, which is often neglected or handled insufficiently. In this paper we aim to explore this problem in more detail and to study the possible solution, which is to evaluate the Fano factor in the operational time. We use equilibrium renewal and Markov renewal processes as spike train models to describe the method in detail, and we provide an illustration on experimental data.

## 1. Introduction

The frequency by which neurons generate spikes (action potentials) is commonly considered as a basic form of information transfer within the neuronal system (Adrian and Zotterman, 1926; Perkel and Bullock, 1968). This “frequency code” is often quantified by the number of spikes within an appropriately set time window. The length of the window is limited by the requirement of stable conditions on one side, and by aiming at reproducible results on the other side. The natural conditions typically vary rapidly, but even if kept constant by an experimenter, there are other reasons, like spiking adaptation (Benda and Herz, 2003), which restrict the duration of the observation time window. All these constrains create difficulties for the statistical inference based on the firing rate, and many sophisticated methods to overcome them have been developed (Nawrot et al., 1999; Dayan and Abbott, 2001; Cunningham et al., 2009; Benedetto et al., 2015; Kostal et al., 2018; Tomar, 2019).

As mentioned, the step from the observed times of spikes to the firing rate quantification requires a proper selection of the statistical procedure. The data available from experiments are usually formed by repeated spike trains (repeated trials) recorded from a single neuron exposed to the identical conditions (reaction to a stimulus). It aims to reflect activity of a neural network of a statistically homogeneous population of neurons. In other words, multiple trials are only an experimental counterpart of real-time multi-neuronal activity. Experiments of multi-unit recordings are also available, however, there is currently no technical tool to simultaneously record all spike trains coming to a target neuron. Independently, whether data come from the repeated experimental trials or from a simultaneous multi-unit neuronal recording, in both cases additional assumptions are often accepted, namely, that the data are independent and statistically identical replicas of the same random variable (which is also assumed in this paper). Then, the common definition of the firing rate is the mean number of spikes per unit time window.

It has been noticed for a long time, and under various experimental conditions, that the firing rate (however defined) does not completely characterize neuronal activity, as demonstrated by many studies going beyond the rate coding concept (Shadlen and Newsome, 1994; Rieke et al., 1999; Olypher et al., 2003; Stein et al., 2005; Kostal et al., 2007; Christodoulou and Cleanthous, 2011; Rajdl et al., 2017). The “neural codes” based on rate-independent components are generally called temporal codes. Probably the simplest one going beyond the mean number of spikes is the variability of that number.

Similar to the firing rate, there are several possibilities on how to quantify the variability of spike trains or in generally non-biological systems (Lindner et al., 2004, 2005; Bravi et al., 2011; Kostal et al., 2013; Aoki et al., 2016; Bowden, 2017; Peterson and Heil, 2018; Ilan, 2020). The variance of the spike count in the observation window would be the typical first candidate. However, large numbers usually have large variance (the second moment is quadratic in the scaling parameter), hence some “relative” dispersion measures are often more desirable. Based on properties of Poisson distribution, as the Poisson process is some kind of template for any series of uniform events appearing in time, the index of dispersion was introduced (Cox and Lewis, 1966). It relates the variance of the number of spikes in a time window to its mean. In Fano (1947) a metric for directly unmeasurable quanta was proposed. It was based on the Poissonian character of the quanta appearance and was also represented as the ratio between variance and mean (Fullagar et al., 2017). It became known as the Fano factor. Despite originally being unrelated to the time evolution and not at all admitting data with a non-Poissonian character, due to the formal equivalence with index of dispersion, the term Fano factor prevailed regardless of the difference in aims. The term Fano factor will therefore be used throughout the paper.

For the Poisson distribution the variance-to-mean ratio is equal to one. This fact has fascinated neuroscientists for many decades and the Fano factor has been evaluated and presented over a wide range of experimental conditions, different types of neurons, and species of experimental animals. The number of references to papers in which it was evaluated would be practically endless. Most of the theoretical models of neurons are oriented toward the calculation of interspike interval (ISI) distribution and directly assume a renewal character of the firing. Many of these studies present coefficient of variation of the interspike intervals. Considering that the squared coefficient of variation is equal to the Fano factor (over an infinite window), experimental studies and also countless numbers of the theoretical papers present the Fano factor as a property of the models investigated in them.

**Figure 1:**
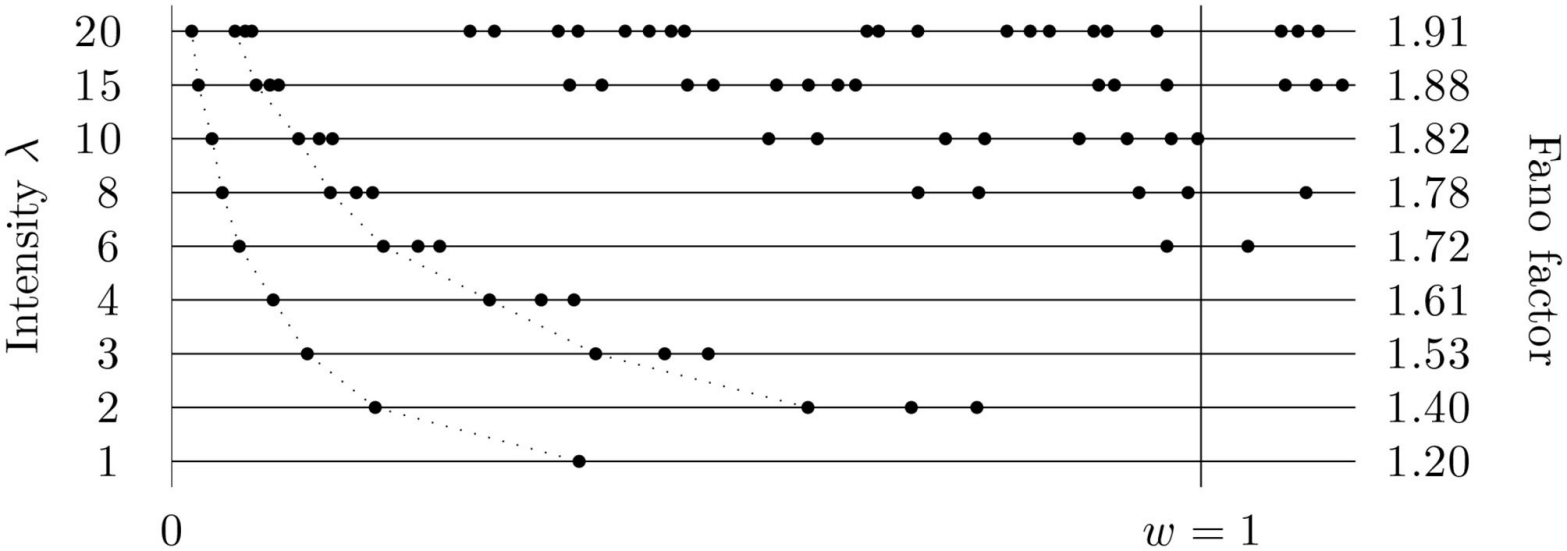
Fano factor might be considered to be an intensity independent measure of variability. This figure (based on simulated data) illustrates that it is not correct; the changes of the intensity influence the (estimated) values of Fano factor significantly. The intensity of the data was changed by scaling time in the original time series and Fano factor calculated in a fixed window of length *w* = 1 (only one, variously scaled, sequence of the times of spikes is shown). The true value of Fano factor (for λ or *w* going to infinity) is 2.

As mentioned above, the mean number of spikes in the observation window is the most common metric for the firing rate. Thus, relating the variance to this quantity may induce the wrong feeling that the measure (index of dispersion, Fano factor) is firing rate independent (Figure 1). Such independence is valid only asymptotically for exceptionally fast firing and exceptionally slow firing, in both cases with respect to the time window over which the counts of spikes are investigated. Neglecting rate changes while studying the Fano factor can lead to incorrect, distorted results, mainly when comparing the Fano factor based on various sets of spike trains from various experiments. A change of the spiking rate changes the estimated values of the Fano factor even if the true values remain constant. This property of the Fano factor is known and there are some ways to avoid this problem, e.g., using the operational time (Nawrot et al., 2008) or the mean matching method (Churchland et al., 2010). However, it seems to us that this issue is still often neglected and has not yet been sufficiently described and explained. For examples of an insufficient Fano factor analysis see the Discussion in Nawrot et al. (2008). Our aim is to describe the dependence of the Fano factor on the intensity in more detail, to show possible problems, and to present and test a method on how to avoid them. The main general solution is to use the Fano factor in the operational time, for the firing rate equal to one. The above-mentioned mean matching method can be suitable too, however, it is usable only in specific situations and is thus not investigated here.

The paper is organized as follows. The relevant theory is summarized in the first part of the paper. We introduce renewal processes and Markov renewal processes, which we use to model the spike trains. Then, the influence of the firing rate on the Fano factor is described and illustrated and a rate-independent variant of the Fano factor is defined. Finally, a method for comparison of variability of two or more data sets with different firing rates is proposed and evaluated.

## 2. Model of Spike Trains

The Fano factor deals with the number of events (spikes) within an observation time window and thus we have to specify the basic properties of such a counting process. The equilibrium counting process *N*(*w*) describes the number of spikes in an interval (0, *w*], *w* > 0, where time zero is “randomly positioned” with respect to the sequence of spikes. The dynamics of the process is captured by its intensity function, λ(*w*), defined by equation

(1) $$\begin{array}{l} \lambda \left(w\right)=\frac{\text{dE}\left(N\left(w\right)\right)}{\text{d}w},w\geq 0, \end{array}$$

which reflects the rate for the occurrence of a new event for a short time window [*w, w* + *dw*). Under the assumption of stationarity, which means that all the statistical properties of *N*(*w*) are time invariant, it holds

(2) $$\begin{array}{l} \text{E}\left(N\left(w\right)\right)=\frac{w}{\text{E}\left(T\right)}\;,w\geq 0\text{,} \end{array}$$

where E(*T*) is the mean of ISIs, which due to the stationarity of the sequence of ISIs is constant, E(*T*) = μ. Combining (1) and (2) we can see,

(3) $$\begin{array}{l} \lambda \left(w\right)=\lambda =\frac{1}{\text{E}\left(T\right)}=\frac{1}{\mu }. \end{array}$$

Unfortunately, formulas analogous to (2) relating higher moments of the counting process to the higher moments of ISIs do not exist. The counting process *N*(*w*) is fully determined by the probabilities *p*_*n*_(*w*) = *P*(*N*(*w*) = *n*)). If *N*(*w*) has Poisson distribution with parameter λ*w*, the process is called the Poisson process. For other commonly investigated point processes there is no analytical expression for *p*_*n*_(*w*).

There is the basic duality relationship between the counting process, *N*(*w*), and intervals between events (ISIs),

(4) $$\begin{array}{l} N\left(w\right)\geq n\Leftrightarrow T_{0}+...+T_{n-1}\leq w, \end{array}$$

where *T*_0_ is time of the first spike and *T*_*n*−1_ is the (*n* − 1)th complete ISI. The spiking process is called the equilibrium renewal process under the additional assumption that the ISIs are independent and identically distributed continuous and positive random variables, denoted by *T*, with the probability density function (pdf) *f*(*t*). The variants usually employed for pdf *f*(*t*) will be summarized later. The term “equilibrium” again specifies that the time zero is unrelated to the sequence of spikes.

Besides the intensity λ, a basic and very often used characteristics of *T* (ISIs) is the coefficient of variation, defined as

(5) $$\begin{array}{l} \text{CV}=\frac{\sqrt{\text{Var}\left(T\right)}}{\text{E}\left(T\right)}, \end{array}$$

where Var(*T*) is the variance of *T*. The main advantage of CV of ISIs, as a measure of spike train variability, is that CV is dimensionless and that ISI probability distributions with different means can often be compared meaningfully. More precisely, CV does not depend on λ if the ISI pdf belongs to the “scale (or rate) family,” i.e., if the pdf *f*(*t*; λ) (explicitly parameterized by the rate λ) satisfies *f*(*t*; λ) = λ*f*(λ*t*; 1) for any λ > 0 (Casella and Berger, 2002). For example, the exponential pdf with fixed refractory period, discussed later in the paper, does not belong to the scale family. Similarly, the value of CV is not changed if the time axis is scaled linearly (e.g., if the refractory period is also rescaled as in our example in section 4).

Renewal processes are standardly used to model neuronal spike trains (Shinomoto et al., 2005; Nawrot et al., 2008; Shimokawa et al., 2010; Omi and Shinomoto, 2011; Ostojic, 2011; Fisch et al., 2012; Pipa et al., 2013; Koyama and Kostal, 2014; Lansky et al., 2016), however, sometimes they might be seen as too simple. We thus also use a generalization of renewal processes–Markov renewal processes (MRPs). In this case the ISIs are not identically distributed, but every ISI has one of the *n* ∈ ℕ probability distributions with pdfs *f*_*i*_(*x*), *i* = 1, …, *n*. The pdfs of the individual ISIs are determined by the states of a Markov process, with a transition matrix *P* = (*p*_*i,j*_), 0 ≤ *p*_*i,j*_ ≤ 1, *i, j* = 1, …, *n*, expressing the probabilities that after an ISI with pdf *f*_*i*_(*x*) there will be an ISI with pdf *f*_*j*_(*x*). Such a process is a combination of a Markov chain with *n* states and a renewal process. Analogously to renewal processes, we assume MRPs in equilibrium. For more detailed description of MRPs see (Çinlar, 1969; Cox and Isham, 1980).

For simplicity, we focus only on a variant with *n* = 2 and *p*_1,2_ = *p*_2,1_ (the probability of transition from the state one to the state two is the same as the probability of reverse transition), thus we have two random variables with pdfs *f*_1_(*x*) and *f*_2_(*x*) (with means μ_1_ and μ_2_ and coefficients of variation CV_1_ and CV_2_) and a transition matrix

(6) $$\begin{array}{l} P=\left(\begin{array}{cc} 1-p & p\\ p & 1-p \end{array}\right), \end{array}$$

where 0 ≤ *p* ≤ 1 is the probability that the state will be changed. The intensity of such a process is

(7) $$\begin{array}{l} \lambda =\frac{2}{\mu_{1}+\mu_{2}}, \end{array}$$

which yields $\lambda /2<\mu_{1}^{-1}\leq \lambda \leq \mu_{2}^{-1}$ (for μ_2_ ≤ μ_1_). If the difference of μ_1_ and μ_2_ is large and *p* is small, we obtain a bursting process. On the contrary, if *p* = 1, the process always changes the states (distributions of ISIs) and is called an alternating renewal process (ARP). A basic example of MRPs (resp. ARPs) is the Markov (resp. alternating) Poisson process (MPP, APP), when the ISIs have exponential distributions $f_{1}\left(x\right)=\mu_{1}^{-1}\exp\left\{-x/\mu_{1}\right\}$ and $f_{2}\left(x\right)=\mu_{2}^{-1}\exp\left\{-x/\mu_{2}\right\},\mu_{1},\mu_{2}>0$.

Finally, let us mention a special and interesting case of equilibrium renewal processes situation for CV → 0. We obtain a sequence of equidistant points (times of spikes) with a random origin. All the ISIs are thus constant with length 1/λ and the only source of variability is the time to the first spike, *T*_0_, which is distributed uniformly. This model is called the (equilibrium) pacemaker (PM) and it represents the limit case of renewal processes from the point of view of (low) variability.

## 3. Fano Factor

Fano factor is a measure of variability of a counting process defined as

(8) $$\begin{array}{l} F\left(w\right)=\frac{\text{Var}\left(N\left(w\right)\right)}{\text{E}\left(N\left(w\right)\right)},\;w>0, \end{array}$$

thus, as the variance to mean ratio of the number of spikes in a time window of a length *w*. As mentioned, this quantity was originally called the index of dispersion for count (Cox and Lewis, 1966). Fano factor defined by (8) is a function of *w*, however, the same term is often used to directly denote the limit

(9) $$\begin{array}{l} F=\underset{w\to \infty }{\lim}F\left(w\right), \end{array}$$

which removes the dependence on *w*.

For renewal processes, basic properties of Fano factor are (Cox, 1962)

(10) $$\begin{array}{l} \underset{w\to 0^{+}}{\lim}F\left(w\right)=1, \end{array}$$

(11) $$\begin{array}{l} F=\underset{w\to \infty }{\lim}F\left(w\right)=\frac{\text{Var}\left(T\right)}{{\text{E}}^{2}\left(T\right)}=\text{C}{\text{V}}^{2}. \end{array}$$

Equation (11) has such an effect that there are cases when CV^2^ is also called Fano factor (Shuai et al., 2002). The function *F*(*w*) cannot be expressed analytically for most of the probability distributions of *T*, however there are some formulas that can be solved numerically, e.g., Rajdl and Lansky (2014),

(12) $$\begin{array}{l} F\left(w\right)=\frac{1}{w}L^{-1}\left\{\frac{1+L\left\{f\right\}\left(s\right)}{s^{2}\left[1-L\left\{f\right\}\left(s\right)\right]}\right\}\left(w\right)-\lambda w, \end{array}$$

where L and $L^{-1}$ denote the Laplace transform and its inversion. For CV → 0 (the equilibrium pacemaker process), we obtain

(13) $$\begin{array}{l} F\left(w\right)=2\tau +1-\frac{\left(\tau +1\right)\tau }{\lambda w}-\lambda w, \end{array}$$

where τ = ⌊λ*w*⌋. The limit of formula (13) for *w* → ∞ is (as expected) zero.

For the Markov renewal processes, analytical calculation of *F*(*w*) is even more complicated. At least the limit of *F*(*w*) for *w* → ∞ can be derived in a closed form, using the results from Ball and Milne (2005),

(14) $$\begin{array}{l} F=\frac{\mu_{2}^{2}\left(2\text{C}{\text{V}}_{1}^{2}-1\right)+2\mu_{1}\mu_{2}+\mu_{1}^{2}\left(2\text{C}{\text{V}}_{2}^{2}-1\right)}{{\left(\mu_{1}+\mu_{2}\right)}^{2}}+\frac{1}{p}\frac{{\left(\mu_{2}-\mu_{1}\right)}^{2}}{{\left(\mu_{1}+\mu_{2}\right)}^{2}}. \end{array}$$

The limit for *w* → 0^+^ equals one, as for the renewal processes. For MPPs relationship (14) reduces to

(15) $$\begin{array}{l} F=1+\frac{1}{p}\frac{{\left(\mu_{2}-\mu_{1}\right)}^{2}}{{\left(\mu_{1}+\mu_{2}\right)}^{2}}, \end{array}$$

and for APPs to

(16) $$\begin{array}{l} F=2\frac{\mu_{1}^{2}+\mu_{2}^{2}}{{\left(\mu_{1}+\mu_{2}\right)}^{2}}. \end{array}$$

The parameter *p* thus highly influences *F*, and by decreasing *p* we can increase *F* arbitrarily. Also, an alternating Poisson process is always more variable than a simple Poisson process; from Equation (16) we can see 1 ≤ *F* < 2, with *F* = 1 only for μ_1_ = μ_2_ (standard Poisson process). Equations (7) and (15) yield that to obtain a MPP with given λ and *F* (for a fixed *p*), the individual means need to be

(17) $$\mu_{1}=\frac{1+\sqrt{p(F-1})}{\lambda },$$

(18) $$\begin{array}{l} \mu_{2}=\frac{2-\lambda \mu_{1}}{\lambda }. \end{array}$$

**Figure 2:**
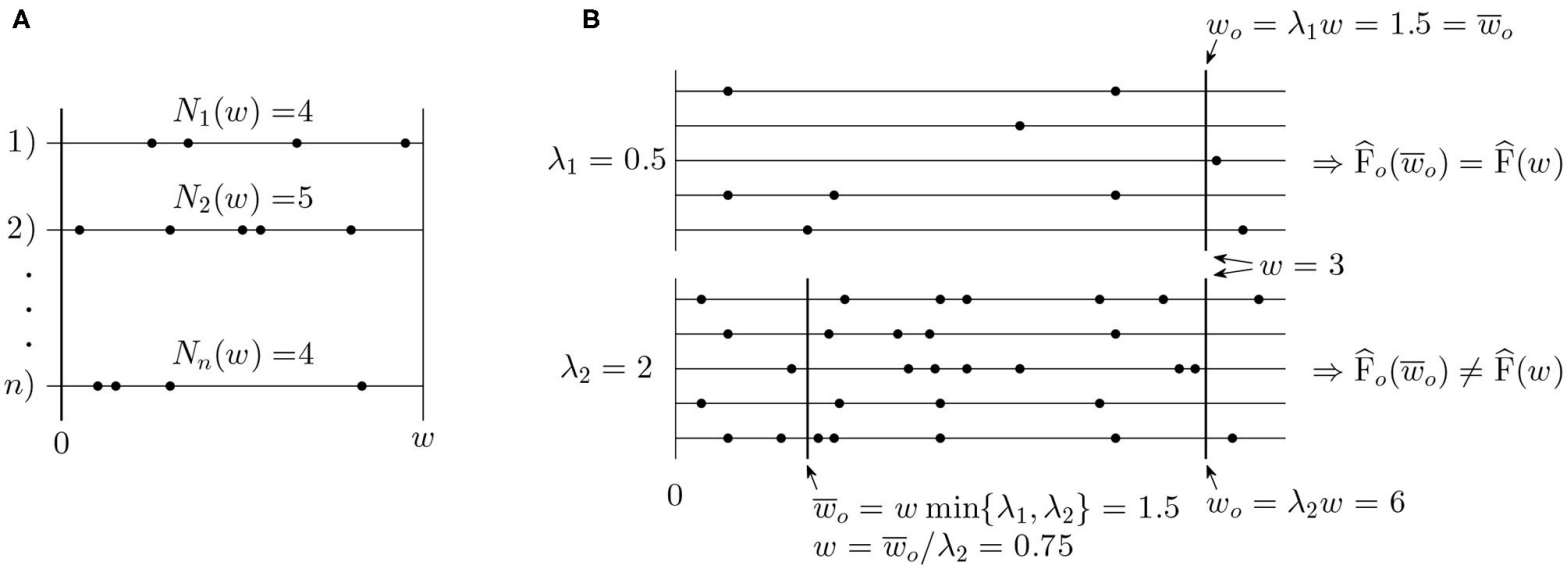
**(A)** Example of typical data used for estimation of the Fano factor. **(B)** Illustration of the operational time. Two sets of spike trains with intensity λ_1_ = 0.5 and λ_2_ = 2 are shown. A window of the original length *w* = 3 has the operational time length *w*_0_ = 1.5 in the first set of spike trains and *w*_0_ = 6 in the second one. For the purpose of the Fano factor comparison, it is more convenient to use the same length of the window in operational time, ${\bar{w}}_{0}$, in both of the cases.

Estimates of the Fano factor, *F*(*w*), and the intensity λ are standardly calculated based on observed numbers of spikes *N*_1_(*w*), *N*_2_(*w*), …, *N*_*n*_(*w*) in a time window (0, *w*], *w* > 0, where *n* is the number of repeated trials or number of simultaneously recorded neurons (see Figure 2A). The standard estimators are

(19) $$\begin{array}{l} \hat{\lambda }=\frac{\bar{N}\left(w\right)}{w}=\frac{1}{nw}\overset{n}{\underset{i=1}{\sum }}N_{i}\left(w\right), \end{array}$$

(20) $$\begin{array}{l} \hat{F}=\hat{F}\left(w\right)=\frac{s_{N\left(w\right)}^{2}}{\bar{N}\left(w\right)}=\frac{\frac{1}{n-1}\sum_{i=1}^{n}{\left(N_{i}\left(w\right)-\frac{1}{n}\sum_{i=1}^{n}N_{i}\left(w\right)\right)}^{2}}{\frac{1}{n}\sum_{i=1}^{n}N_{i}\left(w\right)}. \end{array}$$

Formula (20) is clearly an estimator of *F*(*w*), however, it is used to estimate also directly the limit *F*.

The mean square error (MSE), the common quantity to measure the quality of the estimator, can be decomposed to bias and variance,

(21) $$\begin{array}{l} \text{MSE}\left(\hat{F}\right)=\text{E}{\left(\hat{F}-F\right)}^{2}={\left[\text{E}\left(\hat{F}\right)-F\right]}^{2}+\text{E}{\left[\hat{F}-\text{E}\left(\hat{F}\right)\right]}^{2}\\ \;=\text{Bia}{\text{s}}^{2}\left(\hat{F}\right)+\text{Var}\left(\hat{F}\right). \end{array}$$

The bias and variance of the Fano factor estimator can be approximated as

(22) $$\begin{array}{l} \text{Bias}\left(\hat{F}\right)\approx \left(F\left(w\right)-F\right)+\frac{F\left(w\right)}{\lambda w}-\frac{\text{Cov}\left(s_{N\left(w\right)}^{2},\bar{N}\left(w\right)\right)}{{\left(\lambda w\right)}^{2}}, \end{array}$$

(23) $$\text{Var}(\hat{F})\approx F^{2}(w)\left(\frac{\text{Var}(s_{N(w}^{2}))}{{\text{Var}}^{2}(N(w))}-2\frac{\text{Cov}(s_{N(w)}^{2},\bar{N}(w))}{\lambda w\text{Var}(N(w))}+\frac{F(w)}{\lambda w}\right).$$

Formula (22) shows that the main part of bias of the estimator is unsurprisingly created by the difference *F*(*w*) − *F*.

Besides MSE, a standard measure of error of an estimator is the mean absolute error,

(24) $$\begin{array}{l} \text{MAE}=\text{E}|\hat{F}-F|, \end{array}$$

which is a more natural and better interpretable error measure than MSE, however, it is more difficult to analyze it theoretically.

## 4. Influence of Intensity on Fano Factor

The purpose of the Fano factor evaluation is to measure the variability without the effect of the intensity. However, only the limit value *F* is generally intensity independent. To show this fact, let us consider two identical equilibrium renewal processes *N*_1_(*w*), *N*_2_(*w*), which differ only in the values of intensities λ_1_ > λ_2_ > 0. For this process, it clearly holds

(25) $$\begin{array}{l} P\left(N_{1}\left(w\right)=n\right)=P\left(N_{2}\left(\frac{\lambda_{1}}{\lambda_{2}}w\right)=n\right),\;w>0,n\in {\mathbb{N}}_{0}, \end{array}$$

which directly yields

(26) $$\begin{array}{l} F_{1}\left(w\right)=F_{2}\left(\frac{\lambda_{1}}{\lambda_{2}}w\right),\;w>0. \end{array}$$

The values of the Fano factor for the processes *N*_1_(*w*) and *N*_2_(*w*) are thus generally not equal for all *w* > 0, a change of the intensity changes the value of *F*(*w*) even if all other characteristics are constant.

For an illustration of these facts we show the dependence of *F*(*w*) on the intensity λ for renewal processes with several very often used probability distributions of ISIs, gamma, inverse Gaussian, and shifted exponential. These distributions can be parameterized using λ and *F*, their probability density functions are then

(27) $$\begin{array}{l} f\left(t\right)=\frac{{\left(\lambda /F\right)}^{1/F}t^{\left(1/F-1\right)}e^{-\lambda t/F}}{\Gamma \left(1/F\right)},\;t\geq 0, \end{array}$$

for the gamma distribution, where Γ(*x*), *x* > 0, is the gamma function, and

(28) $$f(t)=\sqrt{\frac{1}{2\pi \lambda Ft^{3}}}e^{-\lambda {\left(t-1/\lambda \right)}^{2})/(2Ft)},\;t\geq 0,$$

for the inverse Gaussian distribution. The most basic distribution of ISIs is the exponential distribution,

(29) $$\begin{array}{l} f\left(t\right)=\lambda e^{-\lambda t},\;t\geq 0, \end{array}$$

yielding the sequences of spikes corresponding to a Poisson process. However, as for this distribution *F*(*w*) = 1 for any *w* > 0, there is no dependence of Fano factor on the intensity. Situation changes when we extend the model for a refractory period (RP) of a length *r* ≥ 0. Refractory period is a time period after each spike when the probability of occurrence of another spike is zero. ISIs then correspond to a random variable *T* = *r* + *T*′, where *T*′ has exponential distribution. If we want such a process to have intensity λ and Fano factor *F*, *F* ≤ 1, its density has to be

(30) $$\begin{array}{l} f\left(t\right)=\left(\lambda /\sqrt{F}\right)e^{-\left(\lambda t-1+\sqrt{F}\right)/\sqrt{F}}\text{ for }t\geq 1/\lambda -\sqrt{F}/\lambda \end{array}$$

and zero otherwise.

To calculate the values of *F*(*w*) we numerically solved formula (12) and confirmed the results by Monte-Carlo method using (20). We also show the values of *F*(*w*) for MPPs with *p* = 1 (APP) and *p* = 0.1 (a bursting process). In these cases, we calculate the means μ_1_ and μ_2_ using Equations (17) and (18) and the values of *F*(*w*) using Monte Carlo simulations. Note that the results for these processes are shown only for *F* = 1.5, as *F* cannot be lower than one. The limit case with zero Fano factor (the pacemaker model) is shown too.

**Figure 3:**
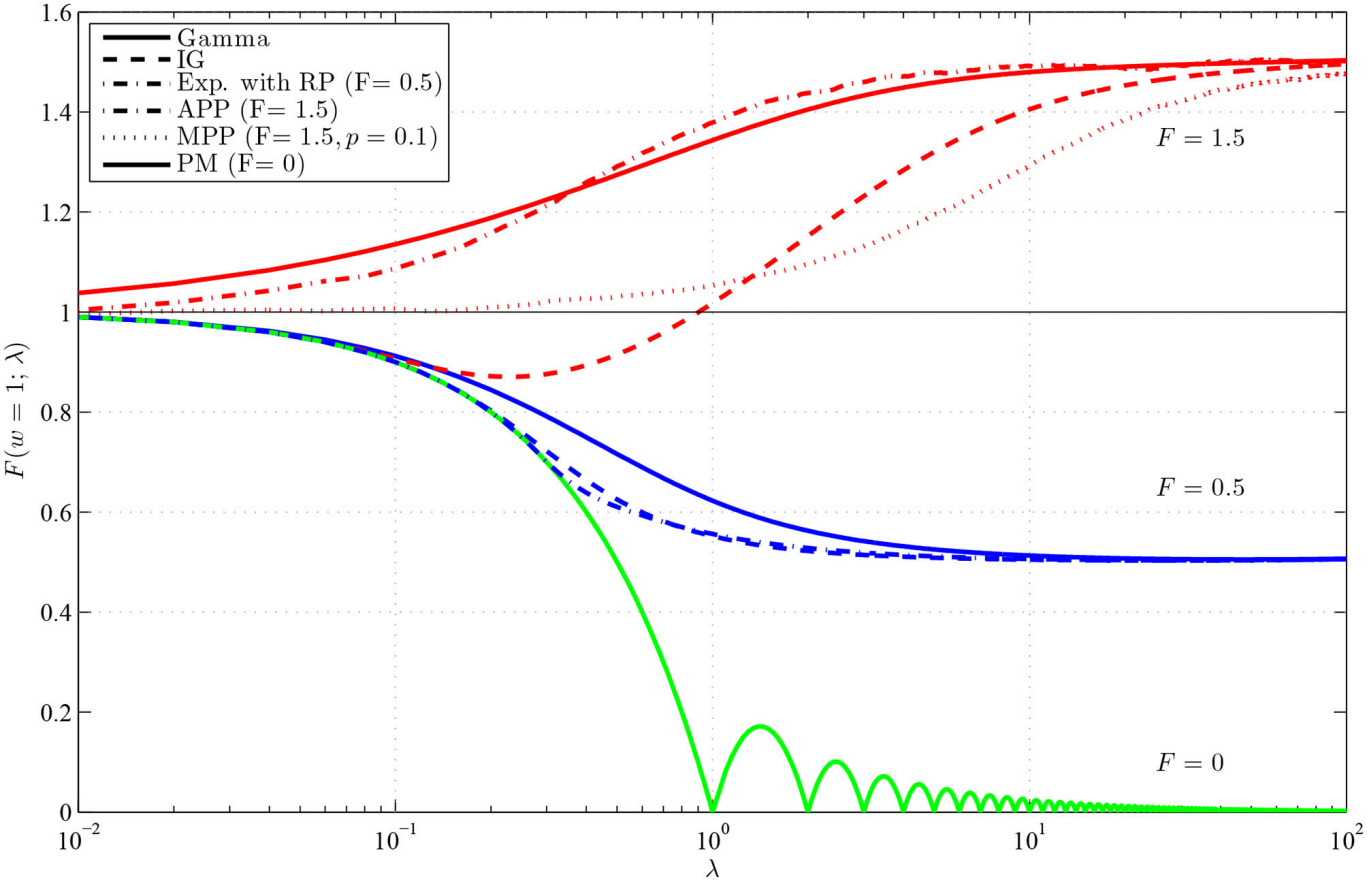
Values of *F*(*w*), for *w* = 1, in dependence on λ for renewal processes with gamma (full lines), inverse Gaussian (dashed lines) and exponential with RP (dashed-dotted line, *F* = 0.5) distributions of ISIs and for Markov Poisson processes with *p* = 1 (APP, dashed-dotted line, *F* = 1.5) and *p* = 0.1 (MPP, dotted line) and for PM process (full line, *F* = 0). The red lines show *F*(*w*) with *F* = 1.5, the blue lines with *F* = 0.5 and the green line with *F* = 0. The λ-axis is logarithmically scaled.

The results are shown in Figure 3, and we can see that the dependence on the intensity is high, yielding various values of *F*(*w*) without changing *w* or *F*. As already mentioned, the dependence on λ is the same as the dependence on *w*, thus we obtained known curves showing the dependence of the Fano factor on the length of the observation window. Note that this holds assuming that the refractory period, included in *T*, scales with the firing rate. If the refractory period is fixed, not influenced by the changes of intensity, the interchangeability of *w* and λ does not hold. There are several interesting features, we would like to point out. Firstly, it is the non-monotonicity of the curve for inverse Gaussian distribution with *F* = 1.5. As shown and explained in Rajdl and Lansky (2014), this holds for any distribution with *f*(0) = 0 and *F* > 1. Secondly, we can see that *F*(*w*) for gamma distribution with *F* = 1.5 converges to unit value for λ → 0 very slowly. It is caused by the fact that probability of occurrence of two spikes in a very short window is in this case not negligible, which results from the specific shape of the gamma distribution with *F* > 1 (the probability of occurrence of a spike near zero is relative large). Finally, for the peacemaker model, the dependence is non-monotonic, with a cyclic-like behavior. Fano factor is zero in integer multiples of the length of the window *w*, when we know exactly how many spikes will be observed.

## 5. Comparison of Fano Factor in Experiments With Various Spike Intensity

It follows from the previous section that the values of *F*(*w*) cannot be studied separately without considering the values of intensity λ. The natural way how to deal with this problem is to compare values of Fano factor only under the condition that the intensity is fixed. Generally, it is suitable to transform time so that the intensity equals one, before calculation (estimation) of *F*(*w*). Such a time (window) is called operational time (Nawrot et al., 2008) and can be expressed as *w*_*o*_ = *wλ*. Two values of Fano factor can be then compared if calculated for the same *w*_*o*_ (see Figure 2B). To avoid prior explicit time transformation, we can define operational Fano factor as

(31) $$\begin{array}{l} F_{o}\left(w_{o}\right)=F\left(w_{o}/\lambda \right)=F\left(w_{o}\mu \right),\;w_{o}>0, \end{array}$$

where *w*_*o*_ is the operational time (time expressed in multiplies of mean ISIs), see Equation (1), but *F*(*w*_*o*_/λ) is calculated on the original time scale. Characteristics *F*_*o*_(*w*_*o*_) is then a variability measure fully independent from the intensity (for equilibrium renewal processes) and it is thus directly comparable even for different experiments.

For estimation of quantity (31) we need to also estimate the intensity. It can be easily done by estimator (19), which yields the estimator of operational Fano factor in form

(32) $$\begin{array}{l} {\hat{F}}_{o}\left(w_{o}\right)=\frac{s_{N\left(w_{o}/\hat{\lambda }\right)}^{2}}{\bar{N}\left(w_{o}/\hat{\lambda }\right)}. \end{array}$$

Measuring Fano factor in operational time is especially important if we want to compare its values estimated based on data from various experiments with various conditions. Nevertheless, there are also some issues complicating this method. Firstly, while estimating the operational Fano factor, we work only with estimated intensities. Estimation of the intensity is a simple task, but it still brings some additional uncertainty (variance) to the value of estimator (32), which could reduce its accuracy. Secondly, we need to determine and use only the greatest common operational time ${\bar{w}}_{o}$ available for all the data (experiments). Suppose that we have *m* > 0 experiments with observation windows *w*_*i*_ and estimated intensities ${\hat{\lambda }}_{i}$, *i* = 1, …, *m*, then

(33) $$\begin{array}{l} {\bar{w}}_{o}=\min\left\{w_{i}{\hat{\lambda }}_{i};i=1,\ldots ,m\right\}, \end{array}$$

see Figure 2B. This implies that some data (spiking times) might not be used, which could seem contra productive. To clarify the severity of these problems and positives of the operational Fano factor while comparing values from different experiments, we perform various evaluations based on Monte Carlo simulations.

In the following we suppose that we have measured the responses, times of spikes fired in a window (0, *w*], of a neuron under two different stimuli, and we are interested in the change of variability in the responses, measured by Fano factor. Thus, we have two sets of *n* > 0 spike trains, first set with intensity λ_1_ and Fano factor *F*_1_ and second with intensity λ_2_ and Fano factor *F*_2_. We aim to find out, how reliable is the ratio *r*(*F*) = *F*_2_/*F*_1_ estimated using the standard estimator (20) and using the estimator of the operational Fano factor (32). Let us denote the estimated ratio for the standard Fano factor as $r\left(\hat{F}\right)$ and for the operational Fano factor as $r\left({\hat{F}}_{o}\right)$.

We study this situation based on data generated using several models of spike trains with various parameters. Specifically, we use equilibrium renewal processes with gamma and inverse Gaussian distribution, MPP with *p* = 0.1 and MPP with *p* = 1 (APP). Without the loss of generality, we fix λ_1_ equal to one and vary λ_2_ in the interval (0, 5]. For the length of the window in operational time we use values 1, 5 and 10 and always generate *n* = 50 spike trains. For *F*_1_ and *F*_2_ we use values 0.5 and 1.5 in all combinations, thus we assume situations when Fano factor increases, decreases and remains constant. Every situation was repeated (generated) 2,000 times.

While using the operational Fano factor, the observation window in the set of spike trains with the larger value of $w\hat{\lambda }$ is shortened. It is thus possible not the use the window which starts at zero, but to shift it within the original window. For example, besides starting at zero, a reasonable possibility could be shifting it to the middle or select its position randomly. This decision depends mainly on where the most likely information is that we want to capture. Nevertheless, while using the equilibrium processes as the spike trains model, the location of the window does not matter. Therefore, in the following examples we use zero as the beginning of the sampling window. Another possibility, how to deal with this question, is to estimate Fano factor repeatedly while shifting the shortened window gradually in the original one and use average as the final estimate. This method also removes the disadvantage of the operational Fano factor, regarding omitting some time of spikes after shortening the window. We evaluate performance of this method too, specifically, we shift the window gradually so that all the spikes are used, but as few as possible spikes are used twice. We denote the corresponding estimator ${\hat{F}}_{õ}$.

**Figure 4:**
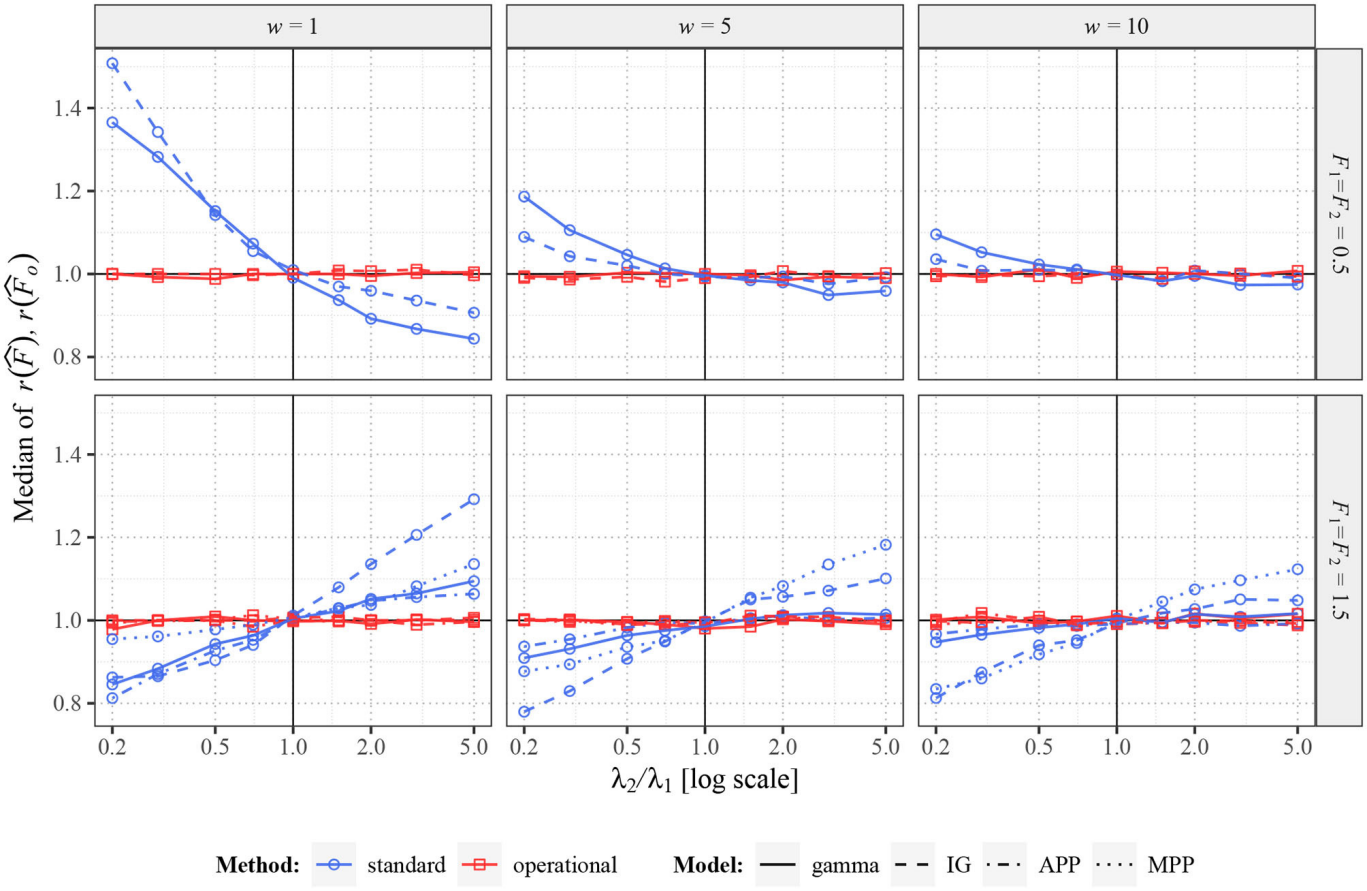
Values of $r\left(\hat{F}\right)$ and $r\left({\hat{F}}_{o}\right)$ for two renewal processes with gamma (full lines) and inverse Gaussian (dashed lines) probability distributions of ISIs and for Markov Poisson processes with *p* = 1 (APP, dashed-dotted line) and *p* = 0.1 (dotted line). Fifty spike trains (*n* = 50) were generated twice with equal values of *F* (*F*_1_ = *F*_2_ = 0.5 in upper panels and *F*_1_ = *F*_2_ = 1.5 in lower panels) and various values of λ_2_ (λ_1_ = 1). Based on this data, estimates of *r*(*F*) were calculated. Three sampling windows (*w* = 1, 5, 10) were used. All the shown values were calculated (generated) 2,000 times and the medians are shown in the graphs. The blue lines (with circles) correspond to the estimates of standard Fano factor, the red lines (with squares) to the estimates of operational Fano factor.

Firstly, in Figure 4, there are shown the median values (across the 2,000 repetitions) of $r\left(\hat{F}\right)$ and $r\left({\hat{F}}_{o}\right)$ in situation when *F*_1_ = *F*_2_. In this scenario, the true ratio of the Fano factor values equals one. While changing intensity λ_2_, the standard Fano factor shows systematically non-unit ratios, thus a false change of variability. The problems are larger mostly for smaller *w*, when the dependence of *F*(*w*) on λ is stronger. Further, the ratios do not tend to unit value for increasing λ_2_, as *F*_1_(*w*) is generally different from *F*_1_ [to which converges *F*_2_(*w*)]. Note that the initial slight non-monotonicity of the curve for the inverse Gaussian distribution in situation with *F*_1_ = *F*_2_ = 1.5 and *w* = 1 is caused by the non-monotonicity of the corresponding curve in Figure 3. Contrary to these inconvenient properties of standard Fano factor, the ratios of the operational Fano factor are always (near) one, the dependence on the intensity is removed. It holds even for MPP with *p* = 0.1, where the dependence of standard Fano factor on the intensity is very high.

**Figure 5:**
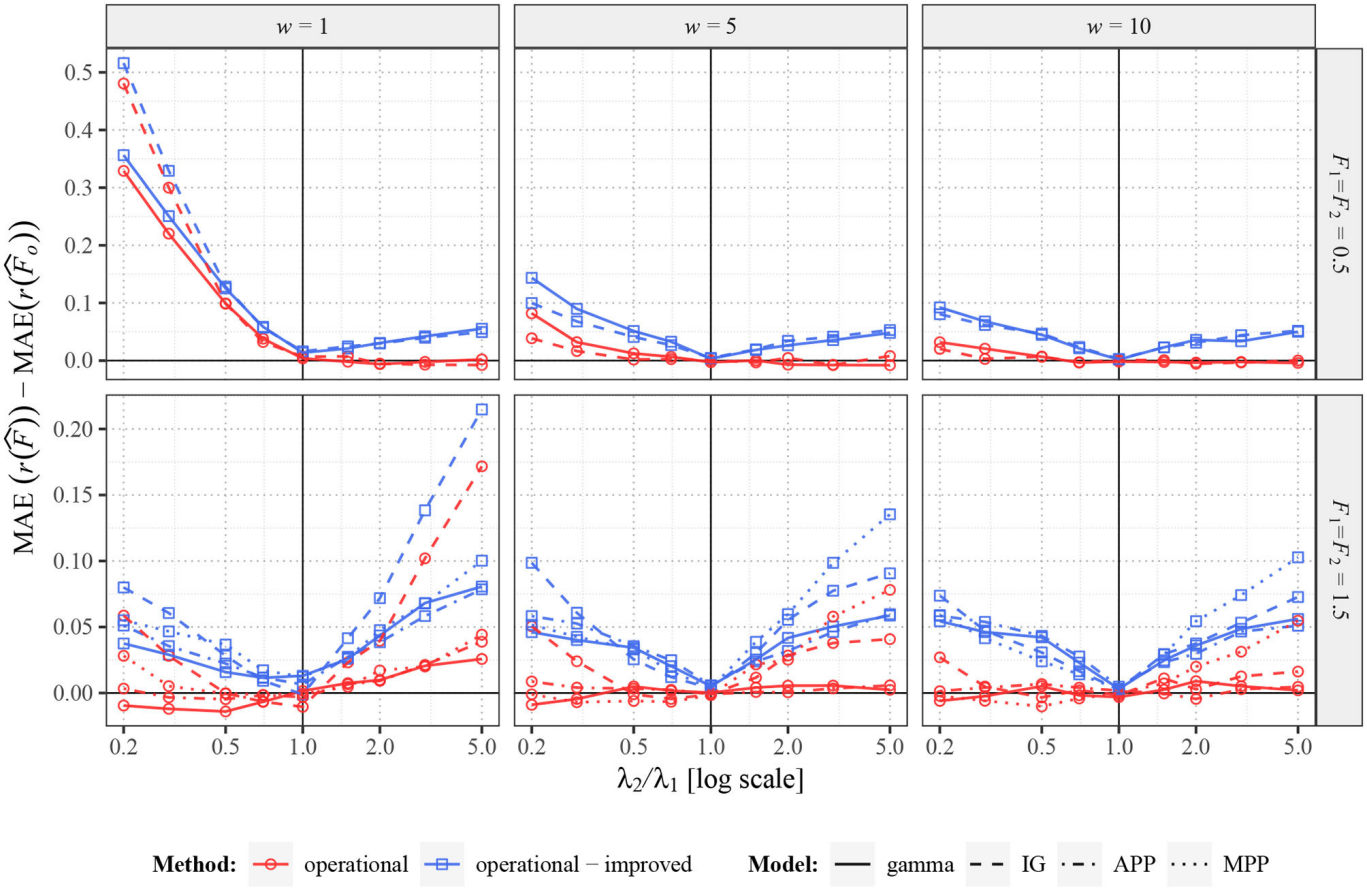
Comparison of MAE of $r\left(\hat{F}\right)$, $r\left({\hat{F}}_{o}\right)$, and $r\left({\hat{F}}_{õ}\right)$ for two renewal processes with gamma (full lines) and inverse Gaussian (MPP, dashed lines) probability distributions of ISIs and for Markov Poisson processes with *p* = 1 (APP, dashed-dotted line) and *p* = 0.1 (MPP, dotted line). Fifty spike trains (*n* = 50) were generated twice with equal values of *F* (*F*_1_ = *F*_2_ = 0.5 in upper panels and *F*_1_ = *F*_2_ = 1.5 in lower panels) and various values of λ_2_ (λ_1_ = 1). Based on this data, MAE of $r\left(\hat{F}\right)$, $r\left({\hat{F}}_{o}\right)$, and $r\left({\hat{F}}_{õ}\right)$ were estimated and differences $\text{MAE}\left(r\left(\hat{F}\right)\right)-\text{MAE}\left(r\left({\hat{F}}_{o}\right)\right)$ and $\text{MAE}\left(r\left(\hat{F}\right)\right)-\text{MAE}\left(r\left({\hat{F}}_{õ}\right)\right)$ for various intensities calculated. Three sampling windows (*w* = 1, 5, 10) were used. All the differences were calculated (generated) 2,000 times and the averages are shown in the graphs.

When the real values of Fano factor remain constant (*F*_1_ = *F*_2_), operational Fano factor fully removes the potentially false change of estimated *r*(*F*), as expected. However, it does not immediately imply, that the operational Fano factor gives better information about *r*(*F*), e.g., the estimator can have larger variance (as explained earlier) and thus possibly also larger MSE or MAE. To explore this possibility we show, in Figure 5, by how much is MAE of the standard estimator larger than MAE of the estimator of the operational Fano factor [assuming one as the real value of *r*(*F*)]. We also show the difference from the improved operational Fano factor $r\left({\hat{F}}_{õ}\right)$. We can see that the operational Fano factor almost always decreases MAE, the few opposite cases are likely just random events. Moreover, the estimator $r\left({\hat{F}}_{õ}\right)$ further decreases MAE significantly.

**Figure 6:**
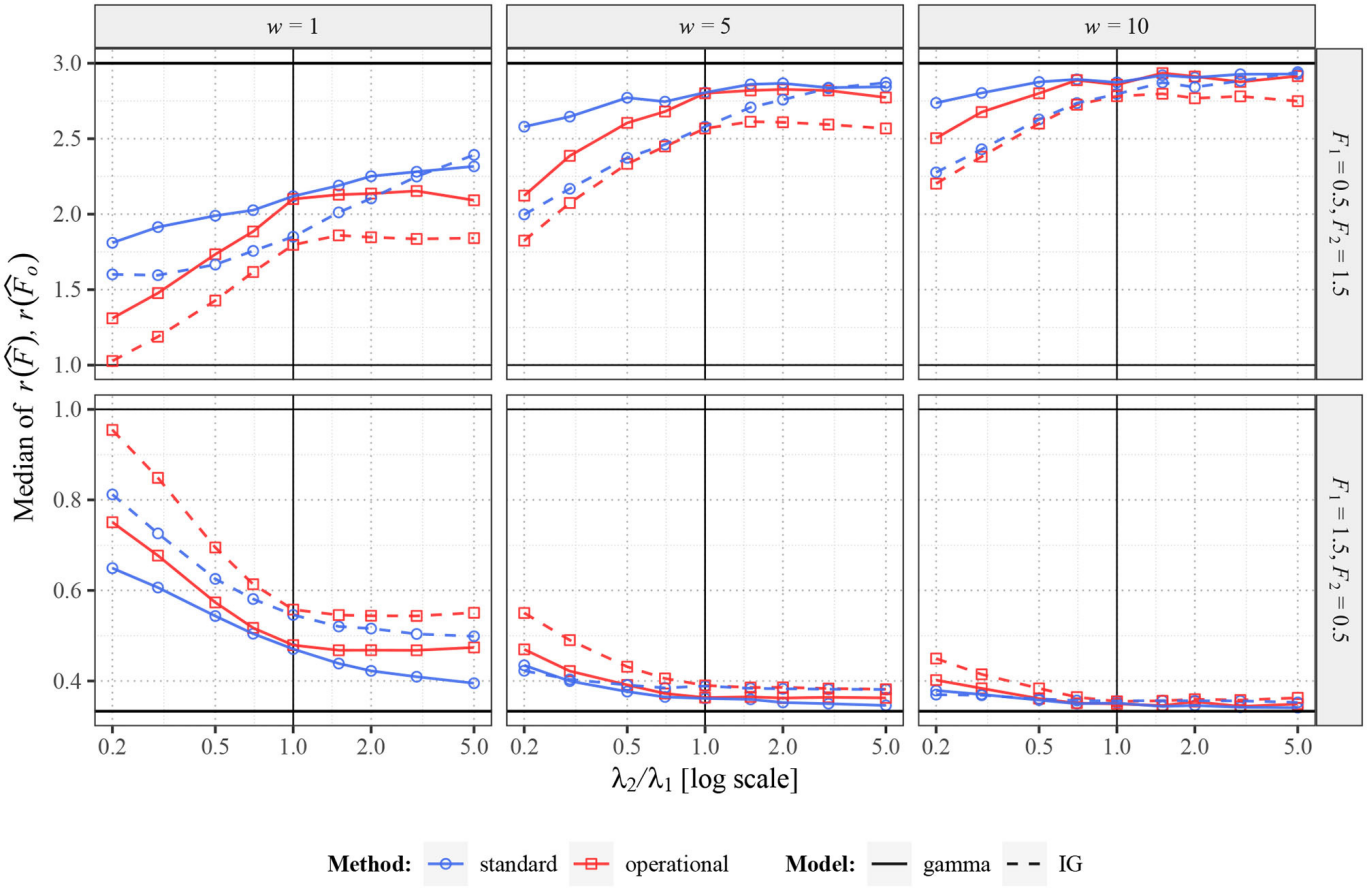
Values of $r\left(\hat{F}\right)$ and $r\left({\hat{F}}_{o}\right)$ for two renewal processes with gamma (full lines) and inverse Gaussian (dashed lines) probability distributions of ISIs. Fifty spike trains (*n* = 50) were generated twice with various values of *F* (*F*_1_ = 0.5, *F*_2_ = 1.5 in upper panels and *F*_1_ = 1.5, *F*_2_ = 0.5 in lower panels) and various values of λ_2_ (λ_1_ = 1). Based on this data, estimates of *r*(*F*) were calculated. Three sampling windows (*w* = 1, 5, 10) were used. All the shown values were calculated (generated) 2,000 times and the averages are shown in the graphs. The blue lines (with circles) correspond to the estimates of standard Fano factor, the red lines (with squares) to the estimates of operational Fano factor.

In Figure 6, there are shown some situations where the values *F*_1_ and *F*_2_ in the individual experiments differ. In this case, it is more complicated to determine the optimal results. Of course, ideally, we would like to obtain value *F*_2_/*F*_1_ independently of λ_2_ as the ratio of the Fano factor estimators, but it is clearly not possible (it would require *w* going to infinity). The best result we can hope for is to obtain constant ratios of Fano factor estimates (independent of λ_2_), positively correlated to *F*_2_/*F*_1_. As we can see, the standard Fano factor does not fulfill this requirement, the differences highly depend on λ_2_ (mainly for small *w*). The behavior of the operational Fano Factor is better, as the ratios are intensity independent at least for λ_2_ > 1. However, for λ_2_ < 1 they change highly even for this variant of Fano factor. It is caused by the fact that decreasing of intensity decreases usable length of the window in operational time, which causes convergence of Fano factor to unit value. The difference from the Figure 4, where shortening of the operational window causes no problems, is that in Figure 4 the bias of the Fano factor estimates (for λ_1_ and λ_2_) due to the short operational window is the same for the both estimates and therefore cancels out in their ratio. The transformation to the operational time removes any bias of the Fano factor estimate caused by differences in intensities, but there is another type of bias due to the limited usage of spikes, which is enhanced when the window is short.

## 6. Real Data Example

**Figure 7:**
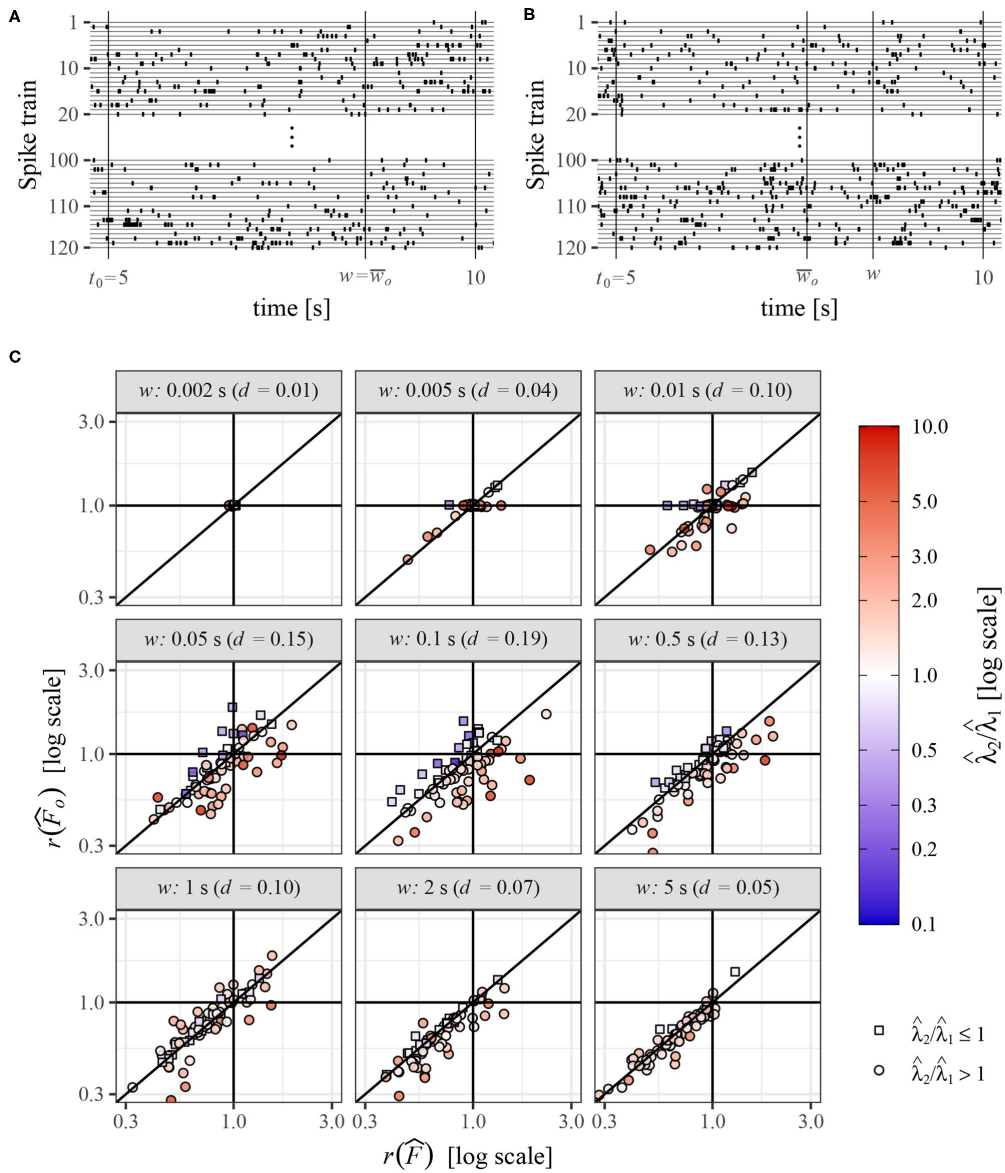
**(A,B)** Example of spike trains from one neuron, for the experiment 1 **(A)** and experiment 2 **(B)**. **(C)** Comparison of changes of variability in two experiments measured by standard Fano factor, $r\left(\hat{F}\right)$, and operational Fano factor, $r\left({\hat{F}}_{o}\right)$, for various length of the observation window *w*. The values were estimated from experimental data, spike trains from 74 neurons recorded under two experimental conditions. Each point corresponds to one neuron and they are distinguished by color and shape according to values of estimated intensities ${\hat{\lambda }}_{1}$ and ${\hat{\lambda }}_{2}$. Value *d* is the mean absolute difference between $r\left(\hat{F}\right)$ and $r\left({\hat{F}}_{o}\right)$.

Similar to the previous section, we study the change of variability between two sets of spike trains but now recorded from real neurons under different experimental conditions. Our aim cannot be to show that information about variability deduced from the operational Fano factor is more accurate than that from the standard method because, in contrast to the simulated data, we do not know the truth. We also do not intend to deduce any specific conclusions about the data, our aim now is only to show that these two methods can yield very different results. The data sets were obtained from www.crcns.org (Kohn and Smith, 2016), where can be found details about the experiment. Briefly, the data consist of spike trains measured in visual cortex of three adult Macaca fascicularis monkeys under three different visual stimuli. While measuring the neuronal activity, the monkeys (anesthetized and paralyzed) were watching three different movies (white noise, a natural scene, and sinusoidal gratings), which yielded spike trains of length 30 s from dozens of neurons. Each experimental setting and measurement were repeated identically 120 times. For our purpose, a much smaller data set is sufficient, we use only a specific part of the data. We compare Fano factor in two of the three experiments, monkeys watching the white noise (experiment 1) and the natural scene (experiment 2). We also use only data of one monkey from time interval 5–10 s as it is sufficient duration for our purpose. This part of data was selected rather randomly as exploring other parts does not yield fundamentally different results. In this data, there are 74 neurons each with 120 repeated spike trains. Based on them we calculate the change of Fano factor in various windows of length *w* and the change using the operational Fano factor concept. The operational Fano factor is calculated in window ${\bar{w}}_{o}$, using formula (33). Thus, we first estimate the intensities in window *w*, ${\hat{\lambda }}_{1}$, and ${\hat{\lambda }}_{2}$, and then shorten the window in the experiment with larger intensity according to the ratio of the intensities. These calculations are performed for each neuron separately—the spike trains from experiments 1 are compared to the spike trains from experiment 2 for the same neuron. An example of the data is shown in Figures 7A,B. Times *w* and ${\bar{w}}_{o}$ illustrate shortening of observation window (*w*) in the spike trains with larger intensity, to obtain the greatest common operational time (window) ${\bar{w}}_{o}$.

This way we obtain (for every *w* and neuron) two estimates of standard Fano factor, ${\hat{F}}_{1}$ and ${\hat{F}}_{2}$, and operational Fano factor, ${\hat{F}}_{o1}$ and ${\hat{F}}_{o2}$. As in the previous section, we measure the change of the estimates as ratios, $r\left(\hat{F}\right)={\hat{F}}_{2}/{\hat{F}}_{1}$ and $r\left({\hat{F}}_{o}\right)={\hat{F}}_{o2}/{\hat{F}}_{o1}$. Note that median values of Fano factors and intensities estimated in the whole window (*w* = 5*s*) are $m\left({\hat{F}}_{1}\right)=6.62,m\left({\hat{F}}_{2}\right)=4.05,m\left({\hat{\lambda }}_{1}\right)=3.48\text{spikes}/s$, and $m\left({\hat{\lambda }}_{2}\right)=4.77\text{spikes}/s$. The values of *F* are relatively high, which is given partially by bursting character and partially by some inhomogeneous behavior of the spike trains in time, resulting from the nature of the experiments (mainly for the movie scenario). This violates the theoretical assumptions, however, as we study the data mostly in short time intervals, the influence should not be serious.

The relation of $r\left({\hat{F}}_{o}\right)$ to $r\left(\hat{F}\right)$ is shown in Figure 7C. The value *d* is calculated as the mean absolute difference between $r\left(\hat{F}\right)$ and $r\left({\hat{F}}_{o}\right)$. We can see that the differences of the results obtained by these two methods can be relatively large, there are even frequent cases when one method shows increase of variability and the other one a decrease [the largest difference is for $r\left(\hat{F}\right)=1.88$ and $r\left({\hat{F}}_{o}\right)=0.78$]. Of course, for small *w* the differences are small (*w* = 0.002 and *w* = 0.005), but they increase with increasing *w* and later decrease again. For *w* = 5 the difference *d* is practically the same as for *w* = 0.005 (although the distributions of the underlying data are different). It corresponds to the theoretical results, for both small and large *w* the influence of intensity on Fano factor decreases and thus the application of the operational Fano factor in these situations does not change the results much. We can also see that for ${\hat{\lambda }}_{1}<{\hat{\lambda }}_{2}$ it is more likely that $r\left(\hat{F_{o}}\right)<r\left(\hat{F}\right)$ and vice versa. This is not a general rule, in this case, it is caused by the fact that the spike trains are mostly highly variable (*F* > 1), which leads to this behavior. The reason is that we are shortening the window in the second experiment and then, from Equation (10), the value of Fano factor mostly decreases nearer to one, decreasing also the ratio $r\left(\hat{F_{o}}\right)$.

## 7. Discussion and Conclusions

The dependency of the Fano factor on firing intensity has been investigated in the previous sections. We studied the effect of the firing rate on the sampling Fano factor *F*(*w*). This approach should not be confused with the study of the objective dependency of the Fano factor, *F*, on the firing intensity, which is described in some experimental studies and in many theoretical models. If an increase in the firing rate is not accompanied by the same increase in the variance of the number of spikes, then the Fano factor *F* changes with the firing rate. For theoretical treatment of this problem, see Koyama (2015), Stevenson (2016), and Charles et al. (2018).

The Fano factor is commonly seen as an analogy to CV based on the numbers of spikes instead of ISIs. They both create two basic measures of variability in series of events. However, the analogy is not accurate. The reason is that the idea of removing the influence of the rate does not work for *F*(*w*) as for CV as the random variable *N*(*w*) depends on linear change of rate (time). The definition (8) is based on a different justification, namely, it is the variance of a counting process related to the variance of Poisson process with the same intensity. For Poisson process the mean count equals its variance and it implies Equation (8). Because of the importance of Poisson process, *F*(*w*) is just a relative variability of the investigated process with respect to the Poisson process. The reason *F*(*w*) became popular as variability measure is its relation to CV for renewal processes (11). However, there is an important difference from CV, the additional parameter—the length of the observation window *w*, which (often highly, mainly for lower *w*) influences the value of *F*(*w*), whereas (theoretical) CV remains constant. Of course, in practice, estimation of CV is also performed for a limited number of ISIs (in other words, in a fixed observation window), leading to similar problems, but of a different nature (caused by censoring of ISIs) (Pawlas and Lansky, 2011; Rajdl and Lansky, 2014). The aim of this paper is to stress that the Fano factor does not parallel the simple notion of CV of ISIs for the case of counts, as often implicitly assumed. We show that even for simple and commonly used renewal models the dependence on λ must be accounted for (i.e., by the method of operational time).

If the Fano factor is employed to measure the variability of spike trains (or other events occurring in time), the effect of the intensity has to be removed. This is not an unknown fact, but it is almost always overlooked or (probably) not entirely understood. We have explored and explained this issue in detail and showed that Fano factor needs to be used carefully. First, we have theoretically explained the influence of intensity on the values of the Fano factor, and illustrated it using several models of neural spike trains. Next, we described how to avoid potential problems - by estimating Fano factor in operational time and defined the intensity-independent approach (the operational Fano factor). Finally, we showed the benefits of the operational Fano factor while comparing the variability in two experiments with different intensities. For the theoretical results and studies based on Monte-Carlo simulations, we used renewal processes and their generalization (Markov renewal processes) as the spike train models.

It is important to emphasize that the theoretical part of the paper was based on specific models of the spike trains, mostly renewal processes. In reality the behavior of neuronal spike trains can be of course much more complex. Let us remind here the main simplifications made when modeling spike trains using the renewal processes, as their violation can cause misinterpretation of the achieved results, for a more detailed discussion see Nawrot et al. (2008). Firstly, the stationarity in time is assumed. This condition can be sufficiently fulfilled in spontaneous activity, but seriously violated in a response to a stimulus. In such a situation, estimation of time dependent intensity and following transformation to operational time can be performed as in Nawrot et al. (2008). However, when the time dependence of intensity is not too significant and the observation window is relatively small (as it mostly is while estimating Fano factor), assuming stationarity in time can be sufficiently accurate. The second possible violation of the theoretical assumptions is non-stability across trials, e.g., when the firing rate in consecutive trials is not constant. This is a troublesome problem, as it is difficult to distinguish between the intrinsic changes and systematic ones. An indicator of this issue can be joint analysis of both *F* and CV, as in such a situation *F* tends to be larger than CV^2^ (Nawrot, 2010). Finally, the renewal assumption is often found violated in real data and it has a large effect on the dependency of the *F* estimate on the length of the observation window, as shown in Chacron et al. (2001), Farkhooi et al. (2009), and Avila-Akerberg and Chacron (2011).

Additionally, to these three potentially critical points we assumed that scaling of time is equivalent to the scaling of intensity, in other words that the ISI distribution belongs to the scale family. It is also a simplification, however, the majority of frequently applied ISI distributions (Gamma and Inverse Gaussian) belong to this family. A warning example when this condition is violated can be consideration of the absolute refractory period, expressed in real (fixed) time. It would imply that the intensity and the observation window are not fully interchangeable and estimating both standard and operational Fano factors, these issues should be kept in mind.

Under the mentioned assumptions, the results can be summarized in the following points.

- Values of Fano factor calculated in a finite window depend on the intensity, even if other characteristics of the spike trains are fixed. Intensity independent (for renewal processes) is only the limit value *F*, the values *F*(*w*) are influenced by λ, mainly for small *w*.
- the influence of the intensity is caused by the fact, that changes of intensity are equivalent to changing the length of the observation window with fixed intensity. Note that this holds under the definition of intensity λ = 1/E(*T*) and *T* from the scale family of distributions. However, for example, in a model with absolute refractory period, the interchangeability of *w* and λ does not hold.
- to remove the influence of the intensity, it is suitable to calculate Fano factor in time linearly transformed so that λ = 1 (operational time) and we show how to calculate an operational Fano factor.
- the influence of intensity has to be taken into account mainly while comparing results from two (or more) experiments. If the intensity is not same, any comparison of Fano factors could be misleading. We show, for example, that a change of intensity causes a false change of estimated Fano factor even if *F* remains fixed.
- This problem can be (mostly) eliminated by using Fano factor calculated in the operational time, when the values are compared for the greatest common operational time.
- If Fano factor is the same between different experiments, the operational Fano factor removes otherwise false change in the observed values. The bias of the differences of the estimated Fano factor is zero and MAE is (in studied cases) smaller than for the standard estimator.
- If the values of Fano factor change between the experiments, the situation is more complex. The operational Fano factor ensures better behavior of the estimates from the point of view of the influence of the intensity, however, it is still necessary to be careful about interpretation of the obtained values even for the operational Fano factor.
- When using operational Fano factor to compare various experiments, some data are wasted. This can be seen as a drawback of the method. It is, however, possible to use all the data so that we shift the shortened window and calculate the estimates of Fano factor multiple times and average them. We show that this procedure further decreases MAE of the estimated differences of Fano factor.

## Data Availability Statement

Publicly available datasets were analyzed in this study. This data can be found at: http://dx.doi.org/10.6080/K0NC5Z4X.

## Author Contributions

PL proposed the concept of the study. KR and PL wrote the manuscript. LK contributed the ideas and suggestions for improvement. KR performed the calculations, numerical simulations, and analysis. All authors contributed to manuscript revision and approved the submitted version.

## Conflict of Interest

The authors declare that the research was conducted in the absence of any commercial or financial relationships that could be construed as a potential conflict of interest.
